# Predictive value of the ^68^Ga-PSMA-11 PET/CT PRIMARY score for pathological upgrading in patients with ISUP Grade 1 prostate cancer and PSA < 20 ng/mL undergoing radical prostatectomy

**DOI:** 10.1007/s12149-026-02179-z

**Published:** 2026-03-03

**Authors:** Esra Ciftci, Burak Akovali, Hatice Sariyildiz Gumusgoz, Burcin Karasah Erkek

**Affiliations:** 1 Faculty of Medicine, Department of Nuclear Medicine, Research and Training Hospital, Sakarya University, Sakarya, Turkey; 2 Faculty of Medicine, Department of Nuclear Medicine, Çanakkale Onsekiz Mart University, Canakkale, Turkey; 3 Department of Nuclear Medicine, Agrı Training and Research Hospital, Agrı, Turkey

**Keywords:** Prostate cancer, ISUP Grade Group 1, ^68^Ga-PSMA-11 PET/CT, PRIMARY score, Pathological upgrading

## Abstract

**Objective:**

Pathological upgrading is common in men with ISUP Grade Group (GG) 1 prostate cancer due to sampling limitations. ^68^Ga-PSMA-11 PET/CT is promising for characterizing tumor biology. The 5-point PRIMARY score, which considers uptake, focality, and location, has limited data on predicting occult high-grade cancer in GG1 biopsy patients. This study assessed the score’s accuracy in predicting upgrading in GG1 prostate cancer patients with PSA < 20 ng/mL undergoing radical prostatectomy.

**Materials and methods:**

This retrospective study included 71 patients with biopsy-proven GG1 prostate adenocarcinoma, PSA < 20 ng/mL, and ^68^Ga-PSMA-11 PET/CT performed within 2 months before radical prostatectomy. PRIMARY scores [1–5] were assigned by two blinded nuclear medicine physicians; scores ≥ 4 were considered high-risk. Pathological upgrading was defined as ISUP GG ≥ 2 on prostatectomy specimens. Diagnostic performance was assessed using ROC analysis, and univariate and multivariate logistic regression models were constructed to determine independent predictors of upgrading.

**Results:**

Pathological upgrading occurred in 45/71 patients (63.4%). Upgrading rates increased markedly across PRIMARY score categories: 25% for scores ≤ 3, 81.3% for score 4, and 100% for score 5 (*p* < 0.001). A PRIMARY score ≥ 4 demonstrated excellent diagnostic performance (AUC = 0.979), with 97.8% sensitivity, 88.5% specificity, 93.6% PPV, 95.6% NPV, and 94.3% accuracy. SUVmax (AUC = 0.944) and PI-RADS ≥ 4 (AUC = 0.721) were also associated with upgrading; however, in multivariate analysis, only the PRIMARY score remained an independent predictor (OR: 19.9; 95% CI: 2.92-135.28; *p* = 0.002).

**Conclusion:**

“The ^68^Ga-PSMA-11 PET/CT PRIMARY score was strongly associated with pathological upgrading in this surgically treated cohort of biopsy-confirmed ISUP GG1 patients with PSA < 20 ng/mL undergoing radical prostatectomy. Incorporating PSMA-based intraprostatic pattern analysis could improve the accuracy of preoperative grading, potentially supporting consideration of surgical management rather than definitive radiotherapy when determining the optimal treatment strategy. Prospective multicenter validation is warranted before broader clinical implementation.

## Introduction

Prostate cancer (PCa) remains one of the most frequently diagnosed malignancies and a leading cause of cancer-related mortality in men worldwide [1]. Accurate risk stratification at the time of diagnosis is the cornerstone of effective management.

Transrectal Ultrasound (TRUS)-guided biopsies sample only a fraction of prostatic tissue, potentially missing aggressive, distinct tumor foci. On the other hand, it is widely accepted that International Society of Urological Pathology (ISUP) grade group (GG) 1 disease is clinically insignificant. Recent systematic reviews indicate that pathological upgrading occurs in a significant proportion of patients initially diagnosed with low-grade disease [2]. According to the landmark study by Ahdoot et al., in patients undergoing radical prostatectomy, systematic biopsy alone undergraded the disease in 41.6% of cases, even with the integration of mpMRI; systematic sampling can miss distinct aggressive foci [3]. Furthermore, although mpMRI has improved detection rates, it still fails to detect clinically significant cancer in approximately 10% of cases and exhibits inter-reader variability [4]. Therefore, accurate noninvasive prediction of upgrading remains a clinical unmet need.

Prostate-Specific Membrane Antigen (PSMA) is a transmembrane protein that is markedly overexpressed in prostate cancer cells. Crucially, PSMA expression levels have been shown to correlate directly with tumor aggressiveness and the Gleason score [5]. While GG 1 tumors typically show low or negligible PSMA avidity, focal intense uptake in these patients may raise suspicion of a coexisting, unsampled high-grade component.

Although quantitative parameters such as SUVmax provide objective data, they can be influenced by physiological variability and may overlap between benign hyperplasia and malignancy. To address these limitations and improve the identification of clinically significant PCa, structured reporting systems, such as the PRIMARY Score, have been developed [6]. It is a five-point categorical scale that integrates signal intensity with specific tracer uptake patterns (e.g., focal vs. diffuse) and anatomical location [6]. Although ISUP grade changes can occur across all ISUP grade groups, accurate preoperative risk assessment is especially important for patients with biopsy-confirmed GG 1 cancer. These patients often undergo active surveillance or watchful waiting, but underestimating tumor aggressiveness could delay necessary treatment. Therefore, reliable noninvasive methods that detect occult, clinically significant prostate cancer in this low-risk group are critically valuable. The ability of this pattern-based scoring system to accurately identify hidden high-grade disease (pathological upgrading) before radical prostatectomy in this ‘low-risk’ setting remains unproven.

The aim of this study is to evaluate the diagnostic performance of the ^68^Ga-PSMA PET/CT PRİMARY score in predicting pathological upgrading among patients with biopsy-proven GG1 PCa and PSA levels < 20 ng/mL, thereby refining risk stratification and guiding more personalized management decisions for this patient cohort.

## Materials and methods

### Study design and patients

Although this study was retrospective, written informed consent was obtained from all patients at the time of 68Ga-PSMA PET/CT imaging. The standardized consent form used at our institution includes explicit permission for the anonymized use of imaging and clinical data for retrospective research, subject to approval by the institutional ethics committee. Therefore, no additional patient re-contact or withdrawal procedure was required for this analysis. The study protocol was approved by the Sakarya University Faculty of Medicine Ethics Committee and conducted in accordance with the principles of the Declaration of Helsinki (decision no: 19.03.2025 – E-43012747-050.04-459120/166).

**Fig. 1:**
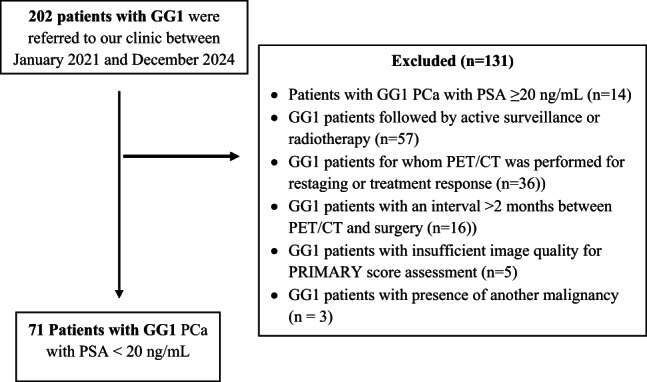
Flow diagram illustrating patient exclusion criteria, and final study cohort. A total of 202 patients with biopsy-proven ISUP Grade Group (GG) 1 prostate cancer were screened. After applying predefined exclusion criteria, 71 patients were included in the final analysis

Between January 2021 and December 2024, 202 patients with biopsy-proven ISUP GG 1 prostate cancer underwent ^68^Ga-PSMA PET/CT. Of these, 131 patients were excluded for the following reasons: GG 1with PSA ≥ 20 ng/mL (*n* = 14), absence of radical prostatectomy (active surveillance or radiotherapy) (*n* = 57), PET/CT performed for restaging or treatment response (*n* = 36), interval > 2 months between PET/CT and surgery (*n* = 16), insufficient image quality for PRIMARY score assessment (*n* = 5), presence of another malignancy (*n* = 3). The remaining 71 patients were further analyzed (Fig. 1). The inclusion criteria were patients with a biopsy-proven GG1 (Gleason Score 3 + 3) prostate adenocarcinoma, preoperative serum Prostate-Specific Antigen (PSA) level < 20 ng/mL, who underwent Radical Prostatectomy (RP), and who had ^68^Ga-PSMA PET/CT imaging within 2 months prior to surgery. A PSA threshold of < 20 ng/mL was used to define a low- to intermediate-risk cohort in whom active surveillance is commonly considered and in whom accurate preoperative prediction of pathological upgrading is most clinically relevant.

### ^68^Ga-PSMA PET/CT study, mpMRI and interpretation criteria

“PET/CT imaging was performed before any treatment on an integrated PET/CT system (Biograph mCT, Siemens Healthineers, Knoxville, USA). All patients underwent imaging approximately 45–50 minutes after intravenous injection of 68Ga-PSMA-11 at a dose of 2–2.5 MBq/kg, in accordance with current EANM guidelines for PSMA PET imaging. Low-dose CT was acquired for attenuation correction and anatomical localization using automated tube current modulation (50–250 mA), tube voltage of 80–120 kV, collimation of 16 × 1.2 mm, pitch of 0.8, and reconstructed with a slice thickness of 5 mm. PET emission data were acquired in 3D mode with an acquisition time of 1.5 minutes per bed position. Images were reconstructed using TrueX + Time-of-Flight (UltraHD-PET) with ordered-subset expectation maximization (OSEM) (2 iterations, 21 subsets), applying a 5-mm Gaussian post-reconstruction filter, into a 256 × 256 matrix. Reconstructed images were transferred in DICOM format to a dedicated workstation (Syngo.via, Siemens Healthineers) for analysis. These standardized acquisition and reconstruction parameters were consistently applied across all patients to ensure reproducibility of the PRIMARY score assessment.

A PRIMARY score using a combination of pattern information and SUVmax was assigned to each patient as defined by *Emmett et al.* [6]. Score 1 was no pattern and low-grade activity. Score 2 was diffuse transition zone (TZ) uptake or symmetric central zone (CZ) activity without focal uptake. Score 3 was focal TZ activity. Score 4 was focal peripheral zone (PZ) activity. Score 5 was any pattern with an SUVmax of at least 12. For patients with multiple patterns, the PRIMARY score represented the most clinically significant pattern (focal pattern above diffuse or symmetric, PZ above TZ, and SUVmax ≥ 12 for any reported pattern) (Table 1). A PRIMARY score of 4–5 was considered positive for high-risk patterns, whereas a PRIMARY score of 1 to 3 was considered indicative of low-risk patterns.

Before the images were evaluated, three nuclear medicine specialists received one hour of training on PRIMARY score evaluation and participated in a consensus read of 20 ^68^Ga-PSMA PET scans external to the study dataset. Two experienced nuclear medicine physicians, blinded to the final pathological outcomes, independently reviewed the images. To reach a single ^68^Ga-PSMA PET imaging decision per patient, any reader disagreement was resolved by a masked read from a third reader, with discrepancies resolved by consensus.

Preoperative PSA values and MRI Prostate Imaging–Reporting and Data System (PI-RADS) scores were retrieved from the hospital’s institutional database. PI-RADS scores were extracted from formal clinical radiology reports generated during routine patient care, and no additional study-specific re-evaluation of mpMRI images was performed. Because multiparametric prostate MRI (mpMRI) was not an inclusion criterion for this study, PI-RADS scores were available only for patients who had undergone mpMRI as part of routine clinical evaluation. Analyses involving PI-RADS were therefore limited to this subgroup. Accordingly, PI-RADS-based analyses were performed in 55 of the 71 patients with available mpMRI data. On mpMRI, the lesion with the highest PI-RADS score and the largest size was considered the index lesion. PI-RADS scores were dichotomized into PI-RADS 4-5 and PI-RADS ≤ 3 groups.

### Histopathological reference standard

The surgical specimens from radical prostatectomy served as the gold standard. Specimens were evaluated by a specialized uropathologist according to the 2014 ISUP consensus. Pathological data were retrieved from the hospital’s institutional database and recorded. Postoperative Gleason scores (GS) were identified as GG1 for GS: 3 + 3, GG2 for GS: 3 + 4, GG3 for GS: 4 + 3, GG4 for GS: 4 + 4, GG5 for GS: 4 + 5, 5 + 4, and 5 + 5. Pathological Upgrading was defined as the presence of ISUP Grade ≥ 2 (Gleason 3 + 4 or higher) in the final prostatectomy specimen.

### Statistical analysis

Statistical analyses were performed using SPSS (Version 24.0; SPSS Inc., Chicago, Illinois, USA). Normality of continuous variables was assessed with the Shapiro–Wilk test. Age showed an approximately normal distribution (*p* > 0.05) and was therefore summarized as mean ± standard deviation, whereas other continuous variables were reported as median (min–max) due to non-normal distributions. Continuous variables (age, PSA, SUVmax) were compared using the Mann-Whitney U test. Categorical variables (PRIMARY Score groups, PI-RADS score) were analyzed with the chi-square test. The diagnostic performance of the PRIMARY score for predicting upgrading was evaluated using receiver operating characteristic (ROC) curve analysis. The area under the curve (AUC), sensitivity, specificity, and the optimal cutoff (Youden index) were obtained. Binary logistic regression was performed to determine whether the PRIMARY score was an independent predictor of upgrading. Candidate variables were chosen based on their clinical relevance. Multicollinearity was checked using variance inflation factors (VIF), and variables with acceptable VIF values were kept in the model. In univariate logistic regression, intraprostatic SUVmax, PRIMARY score, and PI-RADS category were treated as continuous variables to reduce potential information loss from categorization and to assess the stability of effect estimates. Multivariate logistic regression analyses were performed using both Enter and Forward stepwise methods. Model performance was evaluated using odds ratios with 95% confidence intervals, overall classification accuracy, and Nagelkerke’s R² to assess the model’s explanatory power. A p-value < 0.05 was considered statistically significant.

## Results

The mean age of the 71 included patients was 66.4 ± 6.8 years, and all underwent ^68^Ga-PSMA PET/CT for tumor evaluation. Pathological upgrading was observed in 45 patients (63.4%), of whom 34 (47.9%) progressed to GG2 disease, 7 (9.9%) to GG3, and 4 (5.6%) to GG4. The mean PSA level before the prostate biopsy was 9.5 ± 3.3 ng/dL (min-max: 4-19.5). Although the PSA level and the number of positive foci in TRUS biopsy were statistically significantly higher in upgraded patients (*p* = 0.016 and 0.019, respectively), their mean values were similar and not conclusive.

The PRIMARY score of index lesions was Score 1 in 14 (19.7%) patients, Score 2 in 6 (8.5%), Score 3 in 4 (5.6%), Score 4 in 16 (22.5%), and Score 5 in 31 (43.7%). 55 patients underwent Prostat MR imaging, and the PIRADS score of index lesions was PIRADS-2 in 4 (5.6%), PIRADS-3 in 27 (38%), PIRADS-4 in 16 (22.5%), and PIRADS‐5 in 8 (13.5%). Final radical prostatectomy (RP) pathology revealed pT2 disease in 39 patients (54.9%), with 10 (14.1%) classified as T2a, 15 (21.1%) as T2b, and 14 (19.7%) as T2c. Additionally, 26 patients (36.6%) had pT3a, and 6 (8.5%) had T3b. 21 of 26 (81%) patients with T3a disease (extracapsular) at RP were upgraded (15 with GG2, 5 with GG3, 1 with GG4). All 6 patients (100%) with T3b (seminal vesicle invasion) were upgraded (4 with GG2 and 2 with GG4). Only 6 patients had seminal vesicle invasion, and 3 patients had metastasis on 68Ga-PSMA PET/CT (2 in pelvic lymph nodes and 1 in iliac bone), all of whom were among the patients who were upgraded. The baseline characteristics of the upgrading and non-upgrading groups are summarized ***in*** Table 2.

### PRIMARY score and PIRADS score to predict pathological upgrading

ISUP upgrading rates for PRIMARY Score-5, PRIMARY Score‐4, and PRIMARY Score ≤ 3 lesions were 100%, 81.3%, and 25%, respectively (*p* = 0.000). Patients who were upgraded had a significantly higher rate of PRIMARY Score 4–5 lesions than PRIMARY Score ≤ 3 lesions (94% vs. 6.4%, *p* = 0.000) and greater prostatic PSMA uptake (median SUVmax 15.4 vs. 4.5, *p* = 0.000) (Table 2).

The area under the receiver operating characteristic curve (AUC) for the PRIMARY Score to predict pathological upgrading was 0.979 (*p* = 0.000, 95% CI: 0.953–1.000) (Table 3). The optimal cutoff value determined by ROC analysis was 3.5. A PSMA PRIMARY Score of ≥ 4 demonstrated the best discriminative performance for predicting pathological upgrading, with a sensitivity of 97.8%, specificity of 88.5%, positive predictive value of 93.6%, and negative predictive value of 95.6%, yielding an overall accuracy of 94.3%.

When we accept the PRIMARY Score cutoff value as ≥ 3, sensitivity and NPV both increase to 100%, while specificity and PPV decrease to 77% and 88.2%, respectively, with an overall accuracy of 91.5%.

The AUC for intraprostatic SUVmax was 0.944 (*p* = 0.000, 95% CI: 0.896–0.992). SUVmax ≥ 8.1 was identified as the optimal cutoff, with a sensitivity of 82.2%, specificity of 92.3%, positive predictive value of 94.9%, negative predictive value of 75%, and overall accuracy of 86%.

The AUC of the mpMRI PIRADS score applied to 55 patients to predict pathological upgrading was 0.721 (*p* = 0.009, 95% CI: 0.588–0.854), with a cutoff of 3.5. For PIRADS scores of ≥ 4, sensitivity was 57.9%, specificity 88.2%, positive predictive value 91.7%, and negative predictive value 48.4%, with an overall accuracy of 67.2%.

### Univariate and multivariate logistic regression analysis to predict ISUP upgrading

Univariate logistic regression identified several PET/CT and clinical parameters associated with pathological upgrading. The PRIMARY score had the strongest predictive value, with high-risk patterns greatly increasing the likelihood of upgrading (OR = 26.3, 95% CI: 3.45-200.96, *p* < 0.001). SUVmax, as a continuous measure, was also strongly associated, as was the dichotomized variable (OR = 2, 95% CI: 1.38–2.92, *p* < 0.001). Higher PIRADS (OR = 3.25, 95% CI: 1.28–8.24, *p* = 0.004), elevated PSA (OR = 1.26, 95% CI: 1.03–1.55, *p* = 0.02), and more PSMA-positive foci (OR = 1.32, 95% CI: 1.04–1.66, *p* = 0.02) were also significantly associated (Table 4).

Multivariate logistic regression analysis showed that only the PRIMARY score remained independently associated with pathological upgrading (OR = 19.9, 95% CI: 2.92-135.28, *p* = 0.002), with excellent discriminative performance, as indicated by a Nagelkerke R² of 0.720 and an overall classification accuracy of 92.7% (Table 4). SUVmax, PSA level, PIRADS category, and the number of PSMA-positive intraprostatic foci did not retain statistical significance in the adjusted model (all *p* > 0.05).

## Discussion

This study was conducted among patients with biopsy-proven ISUP GG1 disease and PSA < 20 ng/mL. The cohort was not restricted to the D’Amico low-risk category (PSA ≤ 10 ng/mL, GS ≤ 6, clinical stage ≤T2a), because in daily clinical practice GG1 patients often present with heterogeneous or discordant risk features, such as PSA levels > 10 ng/mL or clinical findings suggesting tumor extent beyond T2a, despite a Gleason score of 6 on biopsy. Because treatment selection in this indeterminate subgroup is often uncertain, accurate staging is essential to guide appropriate management. By including these clinically heterogeneous GG1 patients, our study reflects real-world practice and focuses on a subgroup in whom accurate preoperative identification of occult higher-grade disease is particularly relevant. Another important consideration in cohort selection is that pathological upgrading can be confirmed only by histopathological evaluation of radical prostatectomy specimens. Therefore, patients managed with active surveillance or radiotherapy were excluded, as pathological verification would not have been feasible in these groups.

Based on this, in this single-center cohort of 71 men with ISUP GG1 prostate cancer and PSA < 20 ng/mL undergoing radical prostatectomy, we observed a remarkably high rate of pathological upgrading (63.4%), confirming the well-known limitations of TRUS-guided biopsy in accurately assessing tumor aggressiveness. Previous large series have similarly reported upgrading in 14.4–60% of GG1 cases, even with modern imaging and targeted biopsy techniques [3, 7, 8]. These findings highlight the ongoing need for more accurate non-invasive markers for risk stratification in patients with presumed low-risk disease.

The major finding of the present study is that the intraprostatic ^68^Ga-PSMA PET/CT PRIMARY score is a powerful, independent predictor of pathological upgrading in GG1 patients. With a cutoff of ≥ 4, the PRIMARY score showed excellent discriminative performance (AUC = 0.979) and remained the only independent predictor in multivariable analysis (OR = 19.9, *p* = 0.002), outperforming PSA level, SUVmax and the number of PSMA-avid foci. These findings build on the foundational work by Emmett et al., who developed the 5-point PRIMARY scoring system and demonstrated its effectiveness in detecting clinically significant prostate cancer (csPCa) and in enhancing interpretive consistency among readers [6, 9]. In their study, they classified the equivocal score, PRIMARY score 3, as the high-risk group, with a sensitivity of 88%, a specificity of 64%, a PPV of 76%, and an NPV of 81%. In our study, there were only four patients with a PRIMARY score of 3, and only one was upgraded to GG2. Our study provides new insights by specifically examining its role in predicting upgrading in a pure GG1 population, a topic that previous research has not sufficiently explored.

Our findings also align with prior studies showing that PSMA uptake parameters reflect tumor biology. Esen et al. evaluated intraprostatic PSMA uptake in patients with biopsy ISUP 1 undergoing radical prostatectomy and reported that high prostatic PSMA uptake was a very reliable predictor of pathological upgrading, although low uptake did not completely exclude it [10]. A recent review and pooled analyses also suggest that quantitative PSMA parameters (SUVmax, PSMA-derived tumor volume) correlate with higher ISUP grade, adverse pathology and biochemical outcomes [11]. In our cohort, although intraprostatic SUVmax alone was predictive (AUC 0.944; OR 2 for SUVmax), it lost significance in multivariate analysis once the PRIMARY score was included. Although SUVmax is incorporated into the definition of PRIMARY score 5, the PRIMARY score should not be considered a simple categorized form of SUVmax. Unlike a purely quantitative threshold, the PRIMARY scoring system integrates uptake intensity with intraprostatic distribution patterns and zonal anatomy. Clinically significant prostate cancer typically arises in the peripheral zone and tends to demonstrate focal rather than diffuse uptake, whereas benign hyperplasia commonly produces heterogeneous or transition zone–predominant activity. Therefore, the PRIMARY score captures spatial and biological characteristics of tumor behavior that are not reflected by uptake magnitude alone. This likely explains why SUVmax lost significance in multivariable analysis while the pattern-based PRIMARY score remained an independent predictor of upgrading. This supports the concept that the pattern-based PRIMARY scoring system, which integrates uptake intensity, focality and zonal location, captures biologically relevant information beyond a single quantitative threshold and may better discriminate between indolent and clinically significant disease.

The mpMRI PI-RADS score has long been considered a crucial tool for improving prediction, with several studies demonstrating an increased risk of upgrading among men with PI-RADS 4–5 lesions [7, 12]. In our cohort, because mpMRI was available only in a subset of patients, comparisons between the PRIMARY score and PI-RADS were not based on the same population and should therefore be interpreted with caution. PI-RADS ≥ 4 was associated with upgrading (AUC 0.721); however, it did not remain an independent predictor when the PRIMARY score was included in the multivariate model. This aligns with emerging evidence that, although PI-RADS is helpful, a substantial proportion of GG1 patients with PI-RADS 3–4 lesions are still misclassified [7]. Because mpMRI was not available in all patients, a direct paired head-to-head comparison between the PRIMARY score and PI-RADS could not be performed. Therefore, our results should be interpreted as demonstrating complementary information rather than the superiority of one modality over the other.

From a clinical perspective, these findings could influence how patients with GG1 prostate cancer are selected and monitored. Current active surveillance guidelines primarily focus on biopsy grade, PSA levels, and MRI results. Given the high rate of upgrading observed in our GG1 group and the strong association between PRIMARY score ≥ 4 and upgrading, incorporating PRIMARY scoring into decision-making may help identify patients who are not suitable for active surveillance and might benefit from early radical treatment or more aggressive biopsy approaches. Akcay et al. suggested adding the PRIMARY score to active surveillance criteria, arguing that PSMA PET can help avoid surveillance in patients with high-risk intraprostatic patterns [13, 14]. Our results also showed a strong NPV for a PRIMARY score ≤ 3, providing further support for this risk-adapted approach. Similarly, a prospective multicenter study found that combining PSMA PET with MRI improved detection of clinically significant PCa (91% vs. 72%, *p* < 0.001) and increased sensitivity compared with MRI alone (97% vs. 83%, *p* < 0.001), and may reduce the need for confirmatory biopsies in selected patients [15].

We also observed strong associations between upgrading and adverse pathological features, including pT3a–b disease (extracapsular extension and seminal vesicle invasion), consistent with previous reports linking upgrading to more aggressive disease and worse long-term outcomes [8]. Therefore, detecting these patients early is clinically important and may directly influence treatment decisions.

This study has some limitations. Its retrospective design and relatively small sample size may limit generalizability. mpMRI was available for only 55 patients, which may reduce the statistical power for PI-RADS comparisons. Only ^68^Ga-PSMA-11 was used, so applicability to other PSMA tracers remains uncertain. Finally, long-term outcomes were not assessed; thus, although the PRIMARY score predicts upgrading, its impact on recurrence-free or metastasis-free survival remains to be determined. Another limitation is that multiparametric MRI was not available for all patients, so PI-RADS–based analyses were limited to a subset of the cohort, which may have reduced the statistical power of comparisons involving mpMRI. However, mpMRI was not the primary imaging modality or main parameter of interest in this study, which was specifically designed to evaluate the predictive value of the Ga-68 PSMA PET/CT PRIMARY score.

Despite these limitations, our results strongly indicate that the ^68^Ga-PSMA PET/CT PRIMARY score is a reliable imaging biomarker for detecting occult csPCa in men with biopsy GG1 prostate cancer and PSA levels below 20 ng/mL. While limiting inclusion to patients with PSA < 20 ng/mL may reduce PSA variability, this approach mirrors real-world clinical decisions in GG1 cases. In this uniform group, PSA remained significant in univariate analysis but was not an independent predictor in multivariate models, highlighting the superior performance of the PSMA PET/CT PRIMARY score. Although the present study focused on GG1 prostate cancer, the strong association between the PRIMARY score and pathological upgrading suggests that PSMA-based intraprostatic pattern analysis could also help predict grade changes in higher ISUP groups, warranting further research across diverse, multi-grade populations.

Future multi-center prospective studies integrating PRIMARY scoring into risk stratification protocols may help improve patient selection for surgical management instead of definitive radiotherapy when determining the optimal treatment strategy.

## Conclusion

The ^68^Ga-PSMA-11 PET/CT PRIMARY score was strongly associated with pathological upgrading in this surgically treated cohort of biopsy-confirmed ISUP GG1 patients with PSA < 20 ng/mL undergoing radical prostatectomy. A PRIMARY score ≥ 4 was strongly associated with significant disease and was the only independent predictor in multivariate analysis. These results show that PRIMARY scoring adds value beyond PSA, SUVmax, and PI-RADS for preoperative risk assessment. Incorporating PSMA-based intraprostatic pattern analysis could improve the accuracy of preoperative grading, potentially supporting consideration of surgical management rather than definitive radiotherapy when determining the optimal treatment strategy.”

**Table 1:** Definitions of the 5-point PRIMARY score system used in ^68^Ga-PSMA PET/CT

PRIMARY Score	Zone and Pattern	Description
Score 1	-	No pattern or low-grade activity only.
Score 2	TZ (Pattern A)	Diffuse TZ uptake
Score 2	CZ (Pattern B)	Symmetric CZ activity without focal uptake; considered Score 4 if it extends to the peripheral margin.
Score 3	TZ (Pattern C)	Focal activity is at least twice the background TZ uptake.
Score 4	PZ (Pattern D)	Any focal uptake involving the peripheral margin or apex, regardless of intensity.
Score 5	-	Intense uptake with SUVmax > 12.

PZ: peripheral zone; TZ: transition zone; CZ: central zone; SUV: standardized uptake value

**Table 2:** Clinicopathological and imaging characteristics of upgrading and non-upgrading patients

	Upgrade (*n* = 45)	No upgrade (*n* = 26)	*P* value
Age (years) (mean ± SD)	67 ± 6.5	64.7 ± 7.2	> 0.05
PSA at diagnosis, ng/ml (median (min-max))	9.8 (4.4–19.5)	8.2 (4-13.9)	0.016*
Prostate Tumor SUVmax (median (min-max))	15.4 (4-65.4)	4.5 (1.8–9.1)	0.000*
Number of Positive Foci (median (min-max))	5 (1–12)	4 (1–11)	0.02*
*PRIMARY Score*			
Score 1	0	14	< 0.000*
Score 2	0	6	
Score 3	1	3	
Score 4	13	3	
Score 5	31	0	
*ISUP Grade Group*			
GG2 (GS 3 + 4)	34		< 0.000*
GG3 (GS 4 + 3)	7		
GG4 (GS 4 + 4)	7		
*Postoperative T score*			
T2a	2	8	< 0.000*
T2b	5	10	
T2c	11	3	
T3a	21	5	
T3b	6	0	
*mpMRI PIRADS score (n* = 55*)*			
PIRADS ≤ 3	16	15	0.008*
PIRADS 4	14	2	
PIRADS 5	8	0	

GG, ISUP Grade Group; GS: Gleason Score; mpMRI, multiparametric magnetic resonance imaging; PSA, prostate-specific antigen; PIRADS, prostate imaging‐reporting and data system; SUVmax: Maximum of Standardized Uptake Value; SD, standard deviation

*Statistically significant

**Table 3:** ROC curve analysis of parameters in upgrading and non-upgrading patients

PET/CT parameters	Cutoff value	AUC	Sensitivity (%)	Specificity (%)	PPV (%)	NPV (%)	Accuracy (%)	*p* value
PRIMARY Score	4	0.993	97.8	88.5	93.6	95.6	94.3	< 0.001
Tumor SUVmax	8.1	0.992	82.2	92.3	94.9	75	86	< 0.001
PIRADS	4	0.721	57.9	88.2	91.7	48.4	67.2	0.009
PSA level	9	0.672	66.7	61.5	97.3	93.5	95.5	0.02

NPV, Negative predictive Value; PPV, positive predictive value; PSA, prostate-specific antigen; PIRADS, prostate imaging‐reporting and data system; SUVmax: Maximum of Standardized Uptake Value

**Table 4:** Univariate and multivariate logistic regression analysis to predict pathological upgrading

PET/CT parameters	OR	95% CI	*p*-value
*Univariate Analysis*			
PRIMARY Score	26.3	3.45-200.96	< 0.001
SUVmax	2	1.38–2.92	< 0.001
PIRADS	3.25	1.28–8.24	0.004
PSA level	1.26	1.03–1.55	0.02
Number of Positive Foci	1.32	1.04–1.66	0.02
*Multivariate Analysis*			
PRIMARY Score	19.9	2.92-135.28	0.002
